# Pathobiology of an NS1-Truncated H3N2 Swine Influenza Virus Strain in Pigs

**DOI:** 10.1128/jvi.00519-22

**Published:** 2022-05-12

**Authors:** Elien Vandoorn, Wojciech Stadejek, Anna Parys, Sharon Chepkwony, Koen Chiers, Kristien Van Reeth

**Affiliations:** a Laboratory of Virology, Department of Translational Physiology, Infectiology and Public Health, Faculty of Veterinary Medicine, Ghent University, Merelbeke, Belgium; b Laboratory of Veterinary Pathology, Department of Pathobiology, Pharmacology and Zoological Medicine, Faculty of Veterinary Medicine, Ghent University, Merelbeke, Belgium; Hudson Institute of Medical Research

**Keywords:** attenuation, H3N2, influenza A, NS1, pathobiology, swine, type I interferon

## Abstract

Virus strains in the live attenuated influenza vaccine (LAIV) for swine in the United States that was on the market until 2020 encode a truncated nonstructural protein 1 of 126 amino acids (NS1del126). Their attenuation is believed to be due to an impaired ability to counteract the type I interferon (IFN)-mediated antiviral host response. However, this mechanism has been documented only *in vitro* for H3N2 strain A/swine/Texas/4199-2/98 NS1del126 (lvTX98), and several cases of clinical respiratory disease in the field were associated with the LAIV strains. We therefore further examined the pathobiology, including type I IFN induction, of lvTX98 in pigs and compared it with IFN induction in pig kidney-15 (PK-15) cells. lvTX98 induced up to 3-fold-higher type I IFN titers than wild-type TX98 (wtTX98) after inoculation of PK-15 cells at a high multiplicity of infection, while virus replication kinetics were similar. Mean nasal lvTX98 excretion by intranasally inoculated pigs was on average 50 times lower than that for wtTX98 but still reached titers of up to 4.3 log_10_ 50% tissue culture infective doses/mL. After intratracheal inoculation, mean lvTX98 titers in the lower respiratory tract were significantly reduced at 18 to 48 h postinoculation (hpi) but similar to wtTX98 titers at 72 hpi. lvTX98 caused milder clinical signs than wtTX98 but induced comparable levels of microscopic and macroscopic lung lesions, peak neutrophil infiltration, and peak type I IFN. Thus, lvTX98 was partly attenuated in pigs, but this could not be associated with higher type I IFN levels.

**IMPORTANCE** Swine influenza A viruses (swIAVs) with a truncated NS1del126 protein were strongly attenuated in previous laboratory-based safety studies and therefore approved for use as LAIVs for swine in the United States. In the field, however, the LAIV strains were detected in diagnostic samples and could regain a wild-type NS1 via reassortment with endemic swIAVs. This suggests a significant degree of LAIV replication and urges further investigation of the level and mechanism of attenuation of these LAIV strains *in vivo*. Here, we show that H3N2 LAIV strain lvTX98 is only partly attenuated in pigs and is excreted at significant titers after intranasal vaccination. Attenuation and restricted replication of lvTX98 *in vivo* seemed to be associated with the loss of NS1 functions other than type I IFN antagonism. Our findings can help to explain the occurrence of clinical respiratory disease and reassortment events associated with NS1del126-based LAIV strains in the field.

## INTRODUCTION

Influenza A virus (IAV) is a major cause of respiratory disease in pigs and results in significant economic losses in the swine industry. The disease is difficult to control due to the complex swine IAV (swIAV) epidemiology: within the H1 and the H3 subtypes, multiple antigenically different lineages and clades circulate simultaneously (1, 2). swIAVs of each clade continue to evolve due to accumulation of point mutations, which occur mainly in the immunodominant hemagglutinin (HA) surface protein (antigenic drift) (3). In addition, swIAVs can evolve by the exchange of RNA gene segments with other IAVs infecting the same cell (reassortment) (4). Vaccination is the main strategy to control swIAV infections in the field. Most commercially available swIAV vaccines are inactivated. Inactivated vaccines provide sufficient protection against swIAVs that are closely related to the vaccine strains but are less efficient against antigenically distant strains. They induce systemic but no local immune responses. As such, they often protect against virus replication in the lungs without preventing swIAV excretion. In young piglets, their efficacy is often hampered by the presence of maternally derived antibodies (MDA) (5, 6). The live attenuated influenza vaccine (LAIV) that was made available in the United States in 2017, Ingelvac Provenza (Boehringer Ingelheim) (7), can overcome several of these drawbacks. The LAIV can induce a broader immune response, as it elicits a local immune response in addition to systemic antibodies, and can be used in the face of MDA (8–13). However, because of the risk of viral shedding, LAIVs are considered less safe than inactivated vaccines.

The Ingelvac Provenza LAIV contained 2 swIAVs with HA and neuraminidase (NA) surface proteins of A/swine/Texas/4199-2/98 (TX98, H3N2) and A/swine/Minnesota-/37866/99 (MN99, H1N1). Their HAs belong to genetic clades H3 cluster I and H1 γ2-β-like, which no longer circulated when the LAIV became available (14). The 6 internal gene segments of both vaccine strains are derived from TX98, but a deletion in the nonstructural protein 1 (NS1) gene results in the production of a C-terminally truncated NS1 protein consisting of only the first 126 out of 219 amino acids (NS1del126) (15). NS1 is an important virulence factor of IAV. It interferes with host mRNA and protein synthesis, stimulates production of viral proteins, and interacts with many host proteins. An important function is to antagonize the type I interferon (IFN)-mediated antiviral host response (16). The H3N2 LAIV strain TX98 NS1del126 (lvTX98) was impaired in its ability to suppress IFN-α/β induction *in vitro* (15), and this property was associated with strong attenuation *in vivo*. Compared to wild-type TX98 virus (wtTX98), lvTX98 caused only minimal clinical signs and lung lesions. lvTX98 also had a more restricted replication in the swine respiratory tract (15). In addition, shedding of both Ingelvac Provenza LAIV strains was limited after intranasal (i.n.) vaccination of pigs under experimental conditions (10, 17) and in the field (18). These findings suggest that the LAIV strains are safe and pose only a low risk for reassortment events leading to reintroduction of the historic vaccine strains in the field, given their low replication potential. Nevertheless, an increase in H3 cluster I and H1 γ2-β-like swIAVs has been reported in the field in the United States after the licensing of Ingelvac Provenza in 2017 (https://influenza.cvm.iastate.edu/). The HA of these viruses was nearly identical to that of the LAIV strains. Moreover, some of these field strains were reassortants between LAIV and endemic swIAV strains and regained a wild-type NS1 (14). Because the use of Ingelvac Provenza interfered with routine swIAV surveillance in the United States, the vaccine was withdrawn from the market in November 2020 (7).

These field data suggest that NS1del126-based LAIV strains may replicate better in the porcine respiratory tract than assumed based on previous experimental studies. In addition, the kinetics of the type I IFN response after vaccination of pigs with lvTX98 have never been studied *in vivo*. In this study, we further examined the pathobiology of H3N2 LAIV strain lvTX98 and wild-type strain wtTX98 in pigs, including nasal shedding and virus replication in the airways, clinical signs and lung pathology, and type I IFN production.

## RESULTS

### lvTX98 is a stronger IFN-α/β inducer than wtTX98 in porcine epithelial cells.

Restricted lvTX98 replication *in vitro* and *in vivo* was previously linked to an impaired anti-IFN activity *in vitro*, due to its NS1 truncation (15). To confirm this finding, we inoculated pig kidney-15 (PK-15) cells with either lvTX98 or wtTX98 and determined virus and bioactive IFN-α/β titers in supernatants at different hours postinoculation (hpi).

**FIG 1:**
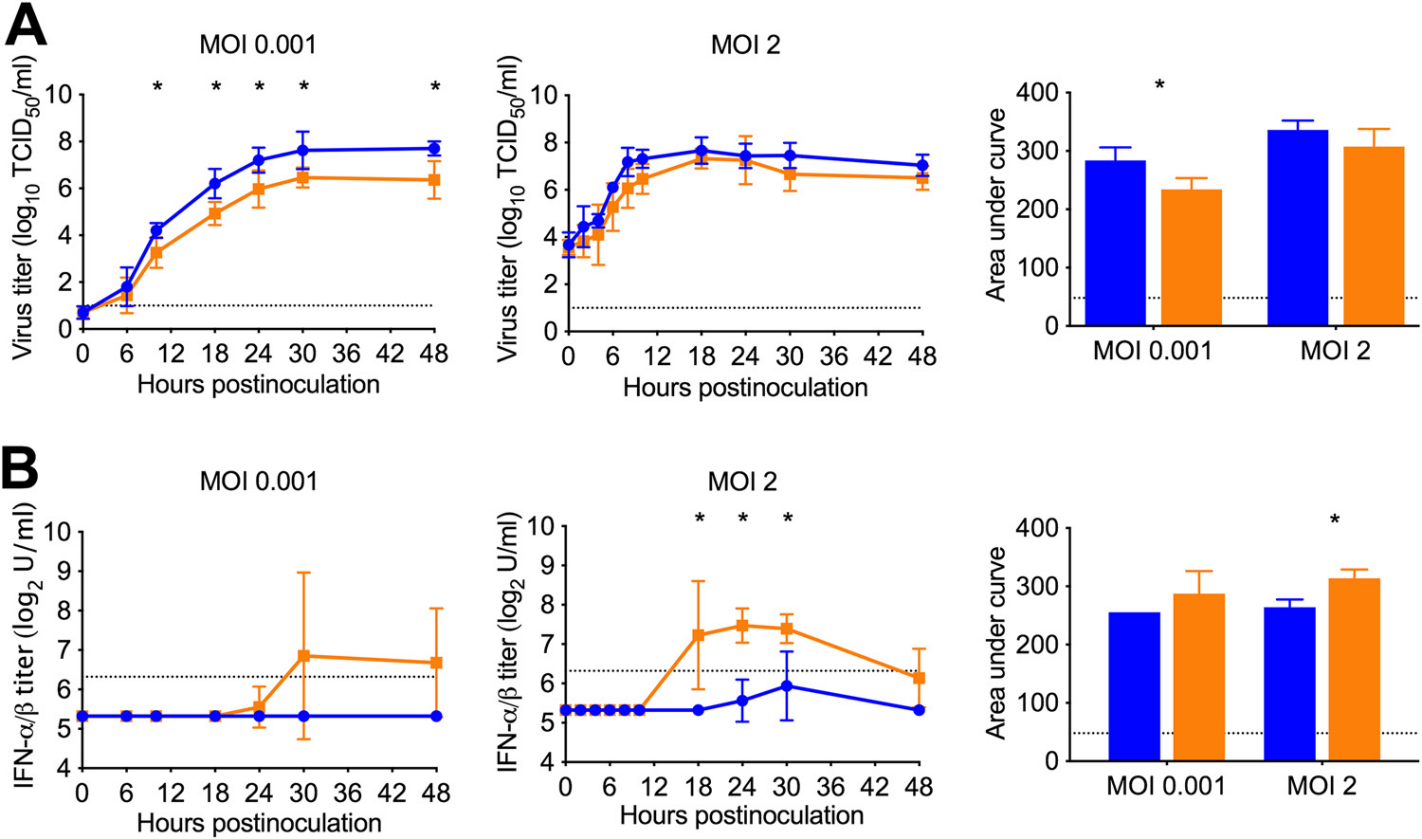
wtTX98 and lvTX98 replication and IFN-α/β-inducing capacity in PK-15 cells. PK-15 cell monolayers were infected with a MOI of 0.001 or a MOI of 2 of wtTX98 (blue circles) or lvTX98 (orange squares), and supernatant samples were evaluated for virus titers in a CPE assay on MDCK cells (A) and for bioactive IFN-α/β titers in a CPE reduction assay with VSV on MDBK cells (B) at different time points postinoculation. Means with standard deviations of log_10_-transformed virus titers, log_2_-transformed IFN-α/β titers, and areas under the curve of 5 replicates are shown. Black dotted lines represent the detection limit. Results were compared between groups using 2-sided Mann-Whitney U tests. *, *P* < 0.05.

wtTX98 replicated to mean titers of up to 7.7 log_10_ 50% tissue culture infective doses (TCID_50_)/mL both at a low multiplicity of infection (MOI) of 0.001 and at a high MOI of 2 (Fig. 1A). Mean lvTX98 virus titers were on average 1.0 log_10_ TCID_50_/mL lower than those of wtTX98 after inoculation at low MOI (*P* < 0.05 at 10 to 48 hpi) but similar to wtTX98 titers after inoculation at high MOI. No bioactive IFN-α/β was detected in PK-15 cell supernatants after inoculation with wtTX98 at a low MOI of 0.001, whereas lvTX98 induced IFN-α/β titers of up to 9.5 log_2_ U/mL in 3/5 replicates of the experiment at this MOI (Fig. 1B). After inoculation at a high MOI of 2, wtTX98 induced detectable bioactive IFN-α/β titers of up to 7.2 log_2_ U/mL in 2/5 replicates of the experiment, while lvTX98 consistently induced IFN-α/β with peak titers that were 3-fold higher than those induced by wtTX98 (*P* < 0.05 at 18 to 30 hpi). Thus, in PK-15 cells, lvTX98 replicates to lower titers than wtTX98 after inoculation at a low MOI and is a better IFN-α/β inducer.

### Reduced nasal excretion of lvTX98 compared to wtTX98 after i.n. inoculation of pigs.

**FIG 2:**
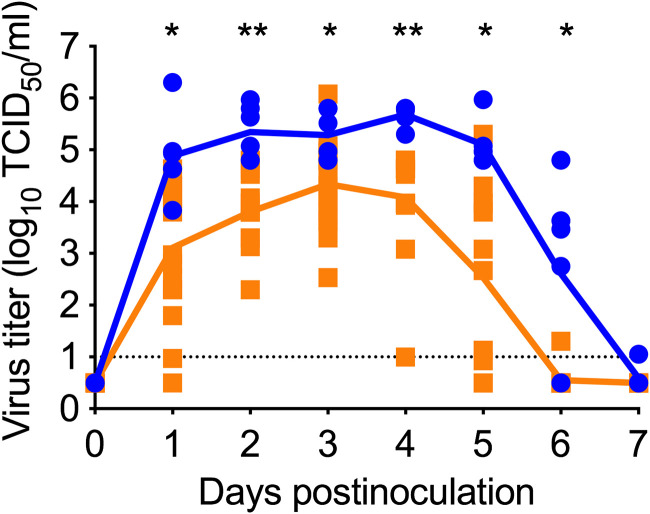
wtTX98 and lvTX98 excretion by intranasally inoculated pigs. Conventional influenza-naive pigs were intranasally inoculated with 6.3 log_10_ TCID_50_ of wtTX98 (blue circles, *n* = 6) or lvTX98 (orange squares, *n* = 16) in 3 mL of PBS, and nasal swabs were taken daily to evaluate virus titers in a CPE assay on MDCK cells. Log_10_-transformed virus titers of individual pigs are shown by dots; lines indicate mean values of each group. Virus titers were compared between groups using 2-sided Mann-Whitney U tests. The black dotted line represents the detection limit. *, *P* < 0.05; **, *P* < 0.001.

In previous studies, i.n. inoculation of pigs with lvTX98 resulted in nasal excretion at mean titers below 1 log_10_ TCID_50_/mL (10), and pigs showed minimal clinical signs (7, 17). However, after commercialization of the Ingelvac Provenza vaccine containing lvTX98, the number of diagnostic field cases associated with the LAIV strains increased (https://influenza.cvm.iastate.edu/). This suggests substantial *in vivo* lvTX98 replication and shedding. To evaluate nasal lvTX98 excretion, pigs were inoculated intranasally with 6.3 log_10_ TCID_50_ of lvTX98 or wtTX98 and clinical signs as well as virus titers in nasal swabs were evaluated at 0 to 7 days postinoculation (dpi). The inoculation dose was selected based on previous vaccination studies with lvTX98 (9–13). None of the pigs showed clinical signs during the experiment. lvTX98 virus could be detected in nasal swabs until 6 dpi, while wtTX98 virus was detected until 7 dpi. Nasal lvTX98 excretion was on average 1.7 log_10_ TCID_50_/mL lower than wtTX98 excretion at 1 to 6 dpi, and the differences were statistically significant (*P* < 0.05, Fig. 2). However, lvTX98 was still excreted at substantial titers: the mean lvTX98 virus titer reached 4.3 log_10_ TCID_50_/mL at 3 dpi. This contrasts with previous studies indicating minimal lvTX98 shedding (10).

### lvTX98 causes milder clinical signs than wtTX98 after i.t. inoculation of pigs.

The considerable lvTX98 excretion after i.n. inoculation of pigs raises questions about the safety of lvTX98. We therefore compared the pathogenicity of this virus in pigs with that of wtTX98. To reproduce the typical clinical signs observed in the field under experimental conditions, pigs were inoculated with a high dose of 7.5 log_10_ TCID_50_ of wtTX98 or lvTX98 via the intratracheal (i.t.) route (5). Pigs were scored for clinical signs from 96 h before inoculation (−96 hpi) until 72 hpi as shown in Table 1. Rectal temperatures and breathing rates were the most important parameters determining the overall clinical score.

**TABLE 1:** Clinical scoring system

Sign	Score	Meaning
Depression/lethargy	0	Reaction to sound
	1	No reaction to sound, reaction to a person entering the stable
	2	No reaction to sound or to a person entering the stable

Tachypnea^*a*^	0	<40 resp./min
	1	40–59 resp./min
	2	60–79 resp./min
	3	≥80 resp./min

Fever	0	Rectal temp 39.0–39.9°C
	1	Rectal temp 40.0–40.9°C
	2	Rectal temp 41.0–41.9°C
	3	Rectal temp ≥42.0°C

Loss of appetite/anorexia	0	Filled stomach
	1	Intermediately filled stomach
	2	Empty stomach

Coughing	0	No
	1	Yes

Dyspnea	0	No
	1	Yes

Labored abdominal breathing	0	No
	1	Yes

Nasal secretion	0	No
	1	Yes

Sneezing	0	No
	1	Yes

Conjunctivitis	0	No
	1	Yes

a Breathing rate per minute was determined for pigs at rest by counting the number of respirations (resp.) per 15 s and then multiplying by 4.

Before inoculation, none of the pigs showed significant clinical signs (Tables 2 and 3). One pig had slight fever at −24 hpi, and some pigs showed occasional tachypnea or sneezing. Coughing, conjunctivitis, and depression were recorded only once. Inoculation with wtTX98 or lvTX98 each led to increased overall clinical scores at 18 to 72 hpi, but peak mean scores were 3 times higher with wtTX98 than with lvTX98.

**TABLE 2:** Prevalence of clinical symptoms after intratracheal inoculation of pigs with wtTX98 or lvTX98

Group	Time point (hpi)	No. of pigs	No. of pigs with:									
			Depression	Tachypnea	Fever	Anorexia	Coughing	Dyspnea	LAB^*a*^	Nasal secretion	Sneezing	Conjunctivitis
wtTX98	−96	15	1	7	0	0	0	0	0	0	0	1
	−72	15	0	5	0	0	0	0	0	0	0	0
	−48	15	0	2	0	0	0	0	0	0	1	0
	−24	15	0	5	1	0	0	0	0	0	1	0
	0	15	0	6	0	0	0	0	0	0	0	0
	18	12	0	10	9	0	0	0	0	0	0	0
	24	9	4	9	8	0	1	0	4	0	1	0
	48	6	1	2	0	0	1	1	1	0	0	0
	72	3	0	3	0	0	0	0	0	0	0	0

lvTX98	−96	15	0	9	0	0	0	0	0	0	2	0
	−72	15	0	3	0	0	1	0	0	0	3	0
	−48	15	0	9	0	0	0	0	0	0	2	0
	−24	15	0	9	0	0	0	0	0	0	5	0
	0	15	0	4	0	0	0	0	0	0	1	0
	18	12	0	8	3	0	0	0	3	0	2	0
	24	9	0	8	1	0	2	0	1	0	1	0
	48	6	1	5	0	0	0	0	0	0	0	0
	72	3	3	1	0	0	0	0	0	0	0	0

a LAB, labored abdominal breathing.

**TABLE 3:** Clinical signs after intratracheal inoculation of pigs with wtTX98 or lvTX98^*a*^

Time point (hpi)	No. of pigs	Rectal temp (°C)		Breathing rate (respirations/min)		Overall clinical score	
		wtTX98	lvTX98	wtTX98	lvTX98	wtTX98	lvTX98
−96	15	39.4 ± 0.2	39.4 ± 0.2	36.1 ± 6.2	38.1 ± 7.7	0.6 ± 0.6	0.7 ± 0.6
−72	15	39.3 ± 0.2	39.2 ± 0.3	35.6 ± 6.9	35.1 ± 6.5	0.3 ± 0.5	0.5 ± 0.5
−48	15	39.1 ± 0.3	39.2 ± 0.2	31.7 ± 6.0	39.3 ± 4.8	0.2 ± 0.4	0.7 ± 0.6
−24	15	39.4 ± 0.4	39.4 ± 0.3	34.3 ± 7.7	38.0 ± 6.5	0.5 ± 0.6	0.9 ± 0.9
0	15	39.2 ± 0.2	39.1 ± 0.3	35.7 ± 9.2	36.5 ± 5.6	0.4 ± 0.5	0.3 ± 0.5
18	12	40.1 ± 0.4	**39.9 ± 0.6**	45.5 ± 8.8	**45.0 ± 10.1**	1.6 ± 0.7	**1.5 ± 1.6**
24	9	**41.0 ± 0.5**	39.6 ± 0.2	**55.1 ± 10.0**	47.1 ± 6.3	**4.2 ± 1.3**	1.4 ± 1.0
48	6	39.1 ± 0.6	39.4 ± 0.3	38.0 ± 16.1	46.3 ± 9.5	1.2 ± 1.2	1.2 ± 0.8
72	3	39.3 ± 0.5	39.1 ± 0.3	45.3 ± 4.6	36.0 ± 10.6	1.0 ± 0.0	1.3 ± 0.6

a Mean values (±SD) are shown; underlined values indicate significant differences between groups at the same time point postinoculation, and boldface indicates significant differences between groups for peak clinical signs (at 24 hpi for wtTX98 versus at 18 hpi for lvTX98) in the 2-sided Mann-Whitney U test (*P* < 0.05).

At 18 hpi with wtTX98, 9/12 pigs had fever with rectal temperatures up to 40.5°C and 10/12 pigs had tachypnea with breathing rates up to 56 respirations (resp.)/min. Clinical signs peaked at 24 hpi: rectal temperatures and breathing rates increased to up to 41.7°C and 64 resp./min, respectively; 8/9 pigs had fever; and all pigs had tachypnea. In addition, several pigs showed labored abdominal breathing, depression, coughing, and sneezing. Fever had disappeared by 48 hpi, but most of the former clinical signs as well as dyspnea and tachypnea were still detected. At 72 hpi, only breathing rates were still elevated.

After inoculation with lvTX98, clinical signs already peaked at 18 hpi. Overall clinical scores, rectal temperatures, and breathing rates were significantly lower than at 24 hpi with wtTX98 (*P* = 0.002, 0.003, and 0.03, respectively). Only 3/12 pigs had fever with temperatures up to 41.4°C, and 8/12 pigs had tachypnea with breathing rates up to 64 resp./min. Some pigs also showed labored abdominal breathing and sneezing. At 24 hpi, these signs as well as coughing were recorded, 1 pig still had fever, and 8/9 pigs had tachypnea. At 48 to 72 hpi, only tachypnea and depression were detected.

Peak clinical signs after i.t. inoculation were thus less abundant and less severe with lvTX98 than with wtTX98. Although lvTX98 could still cause clinical signs, it was attenuated compared to the wild-type virus.

### lvTX98 can induce similar pathological changes in the lower respiratory tract as wtTX98.

At different time points after i.t. inoculation, 3 pigs per group were sacrificed to examine trachea and lung lesions as well as neutrophil infiltration in the lungs caused by lvTX98 and wtTX98. For the latter, cells in bronchoalveolar lavage (BAL) fluid were stained and counted (Table 4).

**TABLE 4:** BAL cells in pigs intratracheally inoculated with wtTX98 or lvTX98

Time point (hpi)	Pig	Total no. of BAL cells (10^6^ cells)		No. of macrophages (10^6^ cells)		No. of neutrophils (10^6^ cells)	
		wtTX98	lvTX98	wtTX98	lvTX98	wtTX98	lvTX98
0	1	319.92	532.90	319.92	525.39	0.00	7.51
	2	105.60	547.60	105.33	546.34	0.27	1.26
	3	697.38	595.43	687.19	593.94	10.18	1.49

18	1	1,194.48	828.18	1,075.03	715.88	119.45	112.30
	2	467.68	599.04	458.37	576.52	9.31	22.52
	3	422.73	904.28	371.37	680.83	51.36	223.45

24	1	1,582.78	944.03	1,381.61	885.13	201.17	58.91
	2	1,094.34	377.41	879.08	351.07	215.26	26.34
	3	1,204.99	394.80	1,028.10	387.26	176.89	7.54

48	1	950.04	962.50	887.34	941.81	62.70	20.69
	2	673.21	933.12	663.31	926.31	9.90	6.81
	3	250.80	542.80	249.77	525.76	1.03	17.04

72	1	720.48	781.44	698.72	771.05	21.76	10.39
	2	446.16	925.60	442.95	919.95	3.21	5.65
	3	499.02	1,198.48	491.78	1,172.83	7.24	25.65

**FIG 3:**
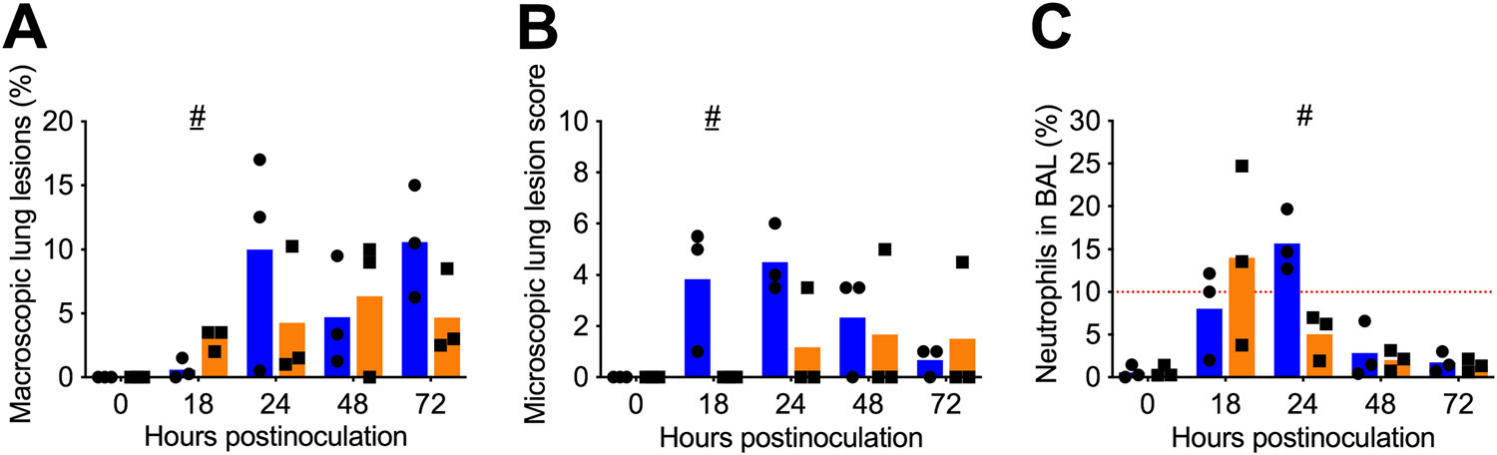
Pathological changes in the respiratory tract of pigs intratracheally inoculated with wtTX98 or lvTX98. Conventional 6-week-old influenza-naive pigs were intratracheally inoculated with 7.5 log_10_ TCID_50_ of wtTX98 (●, *n* = 15) or lvTX98 (■, *n* = 15) in 3 mL of PBS. Three pigs per group were euthanized at different time points. The percentage of macroscopic lung lesions was determined via visual inspection of the lung surface (A); diaphragmatic lung lobe samples were scored for microscopic lesions via histopathological examination (B); BAL cells were isolated from BAL fluid and stained to determine the percentage of neutrophils (C). The red dotted line indicates the threshold for neutrophil infiltration. Dots represent results for individual pigs; blue and orange bars represent mean values for wtTX98- and lvTX98-inoculated pigs, respectively, at each time point. Results were compared between groups using 2-sided Mann-Whitney U tests. #, *P* < 0.1; #, *P* = 0.1.

Macroscopic lesions at the lung surface were detected in both the wtTX98 and the lvTX98 group (Fig. 3A), mainly on the diaphragmatic lung lobes. Gross lung lesions involved up to 17% of the lungs with wtTX98 and up to 10% with lvTX98. Differences between groups were not significant, except at 18 hpi, when on average 2% more macroscopic lung lesions were detected in lvTX98-inoculated pigs (*P* = 0.08).

Microscopic trachea lesions were absent in both groups, except for 1 pig with focal epithelial attenuation at 72 hpi with wtTX98. In the lungs, microscopic lesions were present in 10/12 pigs inoculated with wtTX98 and in only 3/12 pigs inoculated with lvTX98 at 18 to 72 hpi (Fig. 3B and Table 5). wtTX98 caused epithelial damage in up to 75% of the airways, peribronchiolar lymphocytic cuffing in up to 50% of the airways, and minimal to large aggregates of neutrophils in bronchiolar lumens. Similarly, lvTX98 caused epithelial damage in up to 25% of the airways, no to ≥75% peribronchiolar lymphocytic cuffing, and small to large aggregates of neutrophils in bronchiolar lumens of the 3 affected pigs. Only at 18 hpi, composite scores were significantly lower with lvTX98 than with wtTX98 (*P* = 0.06).

**TABLE 5:** Microscopic lung lesions in the diaphragmatic lung lobe of pigs intratracheally inoculated with wtTX98 or lvTX98

Time point (hpi)	Pig	IPAW^*a*^		PBLC^*b*^		Neutro^*c*^	
		wtTX98	lvTX98	wtTX98	lvTX98	wtTX98	lvTX98
0	1	0.0	0.0	0.0	0.0	0	0
	2	0.0	0.0	0.0	0.0	0	0
	3	0.0	0.0	0.0	0.0	0	0

18	1	1.5	0.0	1.5	0.0	2	0
	2	0.0	0.0	1.0	0.0	0	0
	3	1.5	0.0	2.0	0.0	2	0

24	1	2.0	0.0	0.0	0.0	2	0
	2	1.5	0.0	0.0	0.0	2	0
	3	2.5	1.5	1.5	0.0	2	2

48	1	1.5	0.0	1.0	0.0	1	0
	2	0.0	0.0	0.0	0.0	0	0
	3	1.5	1.0	1.0	3.0	1	1

72	1	0.0	0.0	1.0	0.0	0	0
	2	0.0	0.0	1.0	0.0	0	0
	3	0.0	1.0	0.0	1.5	0	2

a Intrapulmonary airway epithelium (IPAW) scores. 0.0, no significant lesions; 1.0, a few airways affected, with bronchiolar epithelial damage; 1.5, more than a few airways affected (up to 25%); 2.0, 50% of airways affected, often with interstitial pneumonia; 2.5, approximately 75% of airways affected, usually with significant interstitial pneumonia; 3.0, more than 75% of airways affected, usually with significant interstitial pneumonia.

b Peribronchiolar lymphocytic cuffing (PBLC) scores. 0.0, no significant lesions; 1.0, a few airways with light PBLC; 1.5, more than a few airways with PBLC (up to 25%); 2.0, 50% of airways with PBLC; 2.5, approximately 75% of airways with PBLC; 3.0, more than 75% of airways with PBLC.

c Scores of neutrophil exudation in bronchioles and alveoli (Neutro). 0, no to minimal presence of neutrophils; 1, small clusters of neutrophils present in occasional airways; 2, prominent small to large aggregates of neutrophils in bronchiolar lumens, with minimal aggregates in alveoli.

Both viruses caused infiltration of neutrophils in the lungs (Fig. 3C). In the wtTX98 group, 2/3 pigs had ≥10% neutrophils in the lungs at 18 hpi. At 24 hpi, a peak in both macrophages and neutrophils was seen for all 3/3 pigs and BAL fluid cells consisted of 13 to 20% neutrophils. In the lvTX98 group, neutrophil infiltration peaked at 18 hpi, when 2/3 pigs had 14 to 25% neutrophils in BAL fluid. Peak percentages of neutrophils in BAL fluid, at 18 hpi with lvTX98 and at 24 hpi with wtTX98, were not significantly different between the two groups (*P* = 0.8).

In summary, lvTX98 was only partly attenuated in terms of lung pathology, as it caused pathological changes less frequently than but at similar severity as wtTX98.

### lvTX98 replicates in both the upper and the lower respiratory tract of pigs.

**FIG 4:**
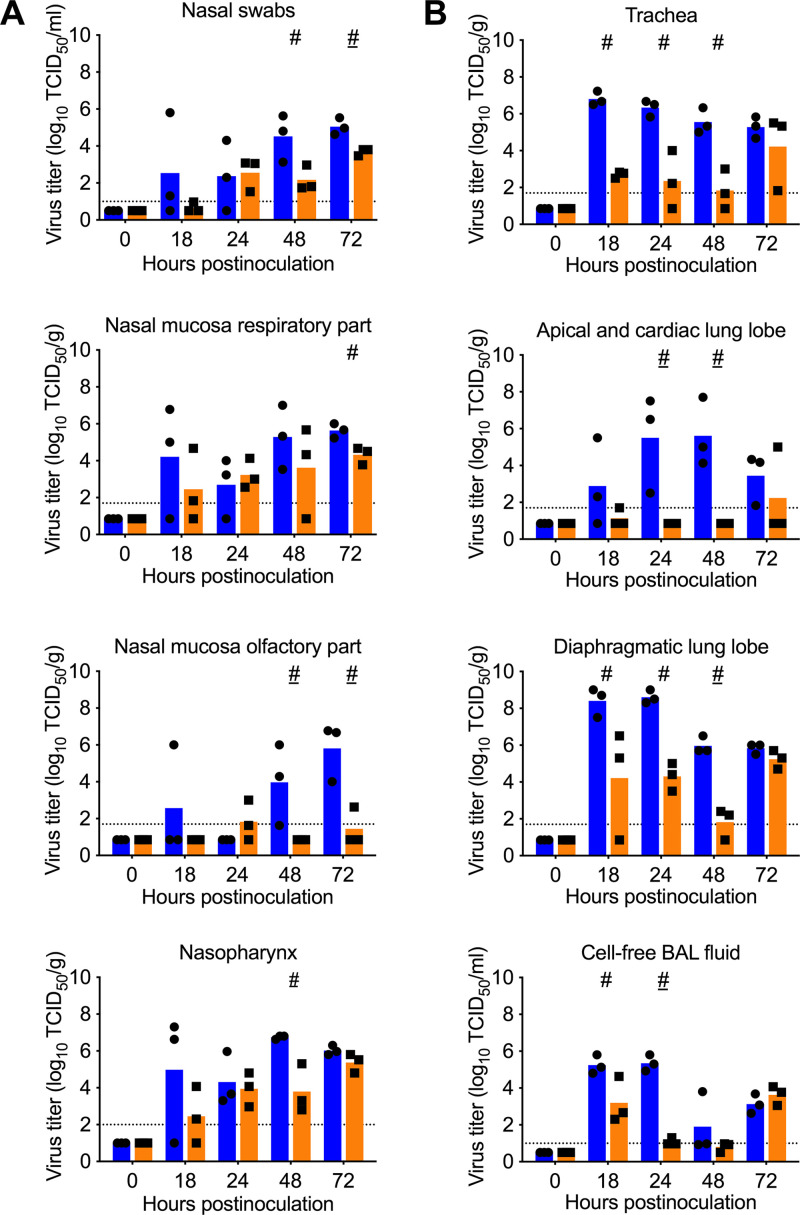
wtTX98 and lvTX98 replication in the respiratory tract of intratracheally inoculated pigs. Conventional 6-week-old influenza-naive pigs were intratracheally inoculated with 7.5 log_10_ TCID_50_ of wtTX98 (●, *n* = 15) or lvTX98 (■, *n* = 15) in 3 mL of PBS. Three pigs per group were euthanized at different time points, and different parts of the respiratory tract were sampled for virus titration via CPE assay on MDCK cells. (A) Upper respiratory tract. (B) Lower respiratory tract. Dots represent results for individual pigs; blue and orange bars represent mean values for wtTX98- and lvTX98-inoculated pigs, respectively, at each time point. Black dotted lines represent the detection limit. Results were compared between groups using 2-sided Mann-Whitney U tests. #, *P* < 0.1; #, *P* = 0.1.

Virus titration was performed on various parts of the respiratory tract collected at different time points after i.t. inoculation. Mean lvTX98 titers were generally lower than mean wtTX98 titers, except at 24 hpi in the nose and at 72 hpi in cell-free BAL fluid (Fig. 4). Variation between pigs was, however, large, and hash marks in Fig. 4 indicate the single time points where all pigs in the lvTX98 group had lower titers than all pigs in the wtTX98 group (*P* ≤ 0.1). Significant differences in the lower respiratory tract were found at 18 to 48 hpi, when mean lvTX98 titers were on average 3.6 log_10_ lower than mean wtTX98 titers (Fig. 4B). In the upper respiratory tract, significant differences were found later, at 48 to 72 hpi, and mean virus titers were on average only 2.2 log_10_ lower for lvTX98 than for wtTX98 (Fig. 4A). Importantly, lvTX98 could replicate to substantial titers of up to 5.8 log_10_ TCID_50_/g in most respiratory tissues. By 72 hpi, lvTX98 titers were comparable to wtTX98 titers in the lower respiratory tract and the nasopharynx. Thus, lvTX98 replication occurred in all parts of the respiratory tract and was not consistently restricted.

### lvTX98 attenuation cannot be related to higher cytokine levels *in vivo*.

lvTX98 has been shown to induce higher levels of IFN-α/β than wtTX98 in the continuous PK-15 cell line, but the kinetics of IFN-α/β secretion in the respiratory tract of pigs have never been examined. Therefore, BAL fluids of pigs inoculated intratracheally with either wtTX98 or lvTX98 were evaluated for levels of IFN-α/β as well as other proinflammatory cytokines, interleukin 6 (IL-6) and tumor necrosis factor α (TNF-α), which were previously shown to be important in swIAV pathogenesis (19).

**FIG 5:**
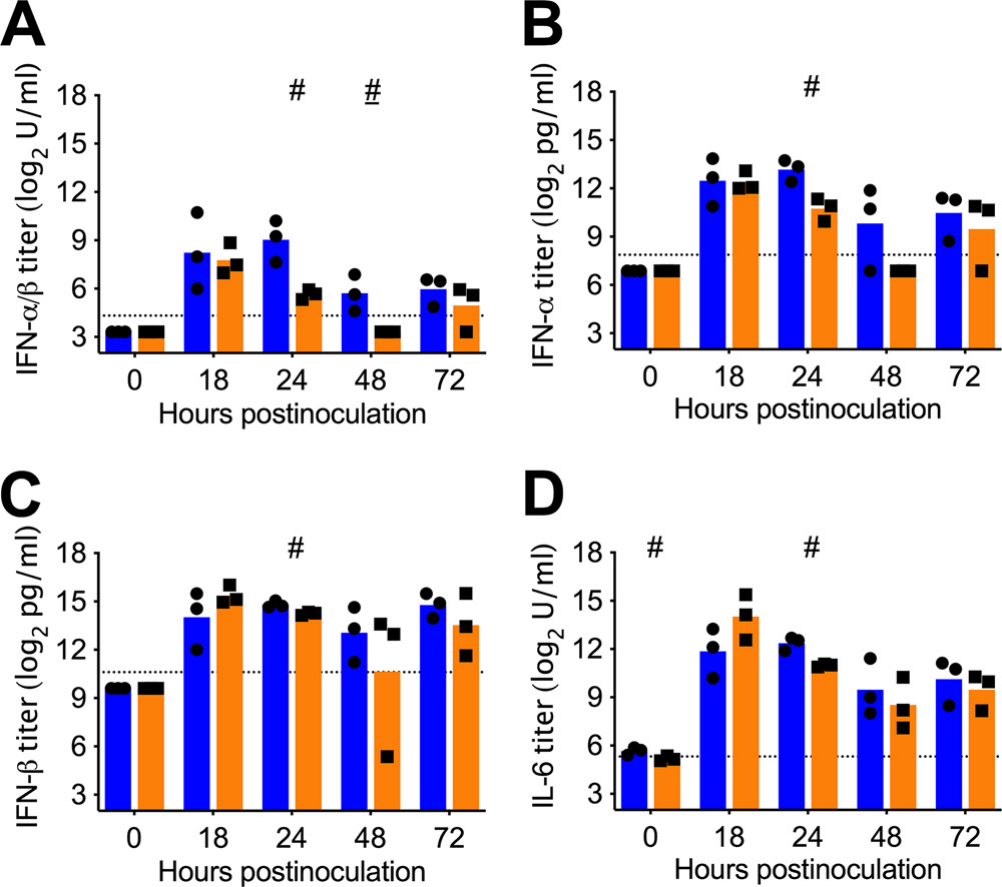
Proinflammatory cytokines in BAL fluid of pigs intratracheally inoculated with wtTX98 or lvTX98. Conventional 6-week-old influenza-naive pigs were intratracheally inoculated with 7.5 log_10_ TCID_50_ of wtTX98 (●, *n* = 15) or lvTX98 (■, *n* = 15) in 3 mL of PBS. Three pigs per group were euthanized at different time points, and BAL fluid was collected. After removal of BAL cells, cell-free BAL fluid was concentrated 20 times for cytokine analysis. (A) Bioactive IFN-α/β titers as determined in a CPE reduction assay with VSV on MDBK cells. (B) IFN-α ELISA titers. (C) IFN-β ELISA titers. (D) Bioactive IL-6 titers as determined in a proliferation assay with B9 cells. Dots represent log_2_-transformed cytokine titers for individual pigs; blue and orange bars represent mean values for wtTX98- and lvTX98-inoculated pigs, respectively, at each time point. Black dotted lines represent the detection limit. Results were compared between groups using 2-sided Mann-Whitney U tests. #, *P* < 0.1; #, *P* = 0.1.

Both wtTX98 and lvTX98 induced IFN-α, IFN-β, and IL-6 in pig lungs. Unlike in PK-15 cells, bioactive IFN-α/β levels in BAL fluids of pigs tended to be lower after inoculation with lvTX98 than after inoculation with wtTX98 (Fig. 5A). The highest IFN-α/β titer in the lvTX98 group was 4 times lower than that in the wtTX98 group. Separate measurements of IFN-α and IFN-β were obtained using enzyme-linked immunosorbent assays (ELISAs). IFN-α ELISA titers reflected the results of the IFN-α/β bioassay and were generally lower in the lvTX98 group than in the wtTX98 group (Fig. 5B). Unlike for IFN-α, the highest IFN-β ELISA titer was detected in a pig of the lvTX98 group (Fig. 5C). Similarly, the highest bioactive IL-6 titer was detected in a pig of the lvTX98 group and was 4 times higher than the highest IL-6 titer in the wtTX98 group (Fig. 5D). However, pigs in the lvTX98 group did not have systematically higher or lower IFN-α, IFN-β, and IL-6 titers than those in the wtTX98 group at the peak of cytokine induction, that is, at 18 hpi for lvTX98 and at 24 hpi for wtTX98. Peak IFN-α, IFN-β, and IL-6 titers were thus not significantly different between the two groups (*P* = 0.2).

lvTX98 and wtTX98 did not consistently induce bioactive TNF-α as measured using a cytotoxicity assay with PK-15 subclone 15 cells. Bioactive TNF-α could be detected in BAL fluid of only 2 pigs in the wtTX98 group (7.1 log_2_ U/mL at 18 hpi and 6.1 log_2_ U/mL at 24 hpi) and 1 pig in the lvTX98 group (5.4 log_2_ U/mL at 18 hpi).

In summary, lvTX98 was not able to induce significantly higher peak levels of IFN-α, IFN-β, IL-6, or TNF-α than wtTX98 in the lungs of i.t.-inoculated pigs. lvTX98 attenuation could therefore not be associated with differential cytokine induction *in vivo*.

### lvTX98 did not obtain additional mutations *in vivo* that were associated with reversion to virulence.

Before the start of the experiments, MinION whole-genome sequencing of the lvTX98 and wtTX98 virus stocks used for inoculation was performed to confirm their genetic constellation. The sequences of the wtTX98 stock were identical to the reference sequences in GenBank, except for synonymous nucleotide mutation G259A in the polymerase acid protein (PA) gene. The NS1 sequence of the lvTX98 stock had a 78-nucleotide (nt) deletion and an insertion of the sequence TAG ATCT TGA T TAA T TAA as previously described (15). The rest of the lvTX98 sequences were identical to the reference sequences in GenBank, except for synonymous nucleotide mutation T1016C in the HA gene and nt mutation A87G resulting in amino acid mutation D27G in the PA gene. Based on analyses in the FluSurver database, the latter was not previously linked to increased virus replication or virulence. Because our *in vivo* results showed less attenuation and higher replication of lvTX98 than previously described, we hypothesized that lvTX98 might have undergone mutations *in vivo* which increased its replication potential and/or virulence compared to the lvTX98 virus used for inoculation. Therefore, whole-genome sequencing was also performed on nasal swabs taken 3 days after i.n. inoculation and on cell-free BAL fluids of pigs sacrificed at 3 days after i.t. inoculation. Viral sequences in these samples were identical to those of the virus used for inoculation. Thus, lvTX98 did not undergo nucleotide or amino acid mutations that could explain its higher replication potential and virulence in this study compared to previous studies.

## DISCUSSION

Our results show that the NS1-truncated H3N2 strain in the LAIV that was available for swine in the United States from 2017 until 2020, lvTX98, is attenuated in pigs: clinical signs were present but milder and lung pathological changes were equally severe as but less frequent than those with wtTX98. This contrasts with the findings of a previous pathogenesis study in which lvTX98 induced no or only minimal clinical signs and lung lesions (15). The latter study, however, used a 220-fold-lower inoculation dose than in our study, which could explain the differences. Although lvTX98 nasal shedding and replication in the swine respiratory tract were restricted compared to those with wtTX98, they were still substantial. Titers in BAL fluids were similar for the two viruses by 3 dpi. In contrast, previous studies showed only minimal lvTX98 shedding after i.n. vaccination (10, 18) and significantly lower lvTX98 titers than wtTX98 titers in BAL fluids at 4 to 5 dpi upon i.t. inoculation (15). These differences might be due to the lower inoculation dose in the latter study, a less sensitive method for evaluating virus shedding in previous studies, and high levels of MDA against lvTX98 in one study (18). We verified that lvTX98 induces higher levels of bioactive type I IFN than wtTX98 in PK-15 cells, although differences between IFN-α/β titers induced by the two swIAVs were smaller than previously reported (15). Our study is the first to show that lvTX98 induces similar levels of IFN-α/β as wtTX98 in swine lungs *in vivo*. Attenuation and restricted replication of lvTX98 in swine are therefore not associated with higher type I IFN induction.

The finding that lvTX98 induces higher levels of IFN-α/β in PK-15 cells *in vitro* but not in swine lungs *in vivo* is likely due to the unsuitability of PK-15 cells as a model for the *in vivo* situation. PK-15 cells most likely synthesize and secrete type I IFN after recognition of viral double-stranded RNA (dsRNA), a by-product of virus replication (20, 21). Although the same IFN pathway may apply in infected airway epithelial cells, the main sources of IFN-α/β during IAV infections *in vivo* are macrophages and, more importantly, plasmacytoid dendritic cells (pDCs) (20–22). Porcine pDCs, also known as natural interferon-producing cells (NIPCs), are probably the most potent IFN-producing cells. They are present at very low numbers in the bloodstream but can migrate to the lungs upon IAV infection and flood the area with type I IFN (20, 21, 23). In contrast to other nucleated cells, NIPCs can be stimulated to rapidly produce large amounts of IFN-α/β by viral glycoprotein structures, independent of viral replication (21). Thus, the majority of IFN-α/β detected in the pig lung is likely produced by cell types and pathways that are not represented in PK-15 cell cultures *in vitro*. Another explanation might be that lvTX98 replication was less restricted in PK-15 cells than in swine. In PK-15 cells, lvTX98 replicated to similar titers as wtTX98 and induced higher levels of IFN-α/β, indicating that lvTX98 has a higher intrinsic IFN-inducing capacity than wtTX98. In swine lungs, lvTX98 replicated to much lower titers than wtTX98 at the time of cytokine induction. As IFN-α/β induction is generally viral dose dependent (24), the lower replication in combination with the higher intrinsic IFN-inducing capacity of lvTX98 may result in the induction of similar IFN-α/β levels as those for wtTX98 *in vivo*. Similarly, a highly virulent mouse-adapted H1N1 IAV with a complete NS1 deletion had a stronger IFN-inducing capacity than the IAV with a partial NS1 deletion *in vitro*, but restricted *in vivo* replication of the former resulted in comparable IFN induction levels by the two IAVs in mouse lungs (25).

Since lvTX98 induces similar IFN-α/β levels as wtTX98 in swine lungs, its attenuation and restricted replication *in vivo* are likely due to the loss of NS1 functions other than antagonism of the type I IFN response. Indeed, type I IFN in the lungs of IAV-infected pigs positively correlates with both virus titers and clinical signs (19), indicating a role in disease rather than attenuation. In addition, NS1 does not seem to be a potent inhibitor of type I IFN induction, as evidenced by the high IFN-α/β levels in the lungs of wtTX98-infected pigs. NS1del126 protein levels in PK-15 cells after lvTX98 infection were much lower than NS1 protein levels after wtTX98 infection (15) and the NS1del126 protein of mouse-adapted strains, which was sufficient to suppress IFN-β induction *in vitro* (26, 27), was unstable *in vivo* due to the lack of the C-terminal domain (27). Therefore, the NS1del126 protein of lvTX98 is likely unstable and thus present at only minimal levels both *in vitro* and *in vivo*. Since IAVs lacking NS1 replicated about 100 times less well than the parental virus, even in Vero cells that are unable to produce IFN (24, 28, 29), NS1 functions other than interference with the type I IFN system of the host are important for viral replication and virulence. Attenuation and restricted replication of lvTX98 in pigs may be due to the elimination of NS1 effects such as inhibition of host protein synthesis as well as stimulation of viral RNA polymerase and translation of viral proteins (16).

Attenuation and immunogenicity of NS1del126 IAVs such as lvTX98 were previously observed in mice, ferrets, poultry, horses, macaques, and pigs, and they were therefore considered suitable LAIV candidates (8, 30–32). However, lvTX98 attenuation is obtained by alteration of only 1 gene segment, allowing the virus to regain a wild-type gene constellation via reassortment with IAVs that are endemic in the vaccinated swine herd. Since reassortment is facilitated by high virus replication, and based on the lvTX98 virus titers observed in this study, lvTX98 might not be sufficiently attenuated to use as an LAIV strain in the field. Indeed, after Ingelvac Provenza was licensed in the United States in 2017, the diagnostic cases submitted to the Iowa State University Veterinary Diagnostic Laboratory with an HA related to vaccine strains lvTX98 (H3N2) and lvMN99 (H1N1) increased to 3.946% and 2.857% in 2019, respectively, compared to <0.1% in 2016. The number of cases again decreased to <1% in 2020 to 2021, after withdrawal of the Ingelvac Provenza vaccine from the market (https://influenza.cvm.iastate.edu/) (33). Clinical signs and lung lesions consistent with IAV infection were reported for these cases, although a role for viral and/or bacterial coinfections could not be excluded (14). Reassortment between LAIV and endemic IAV strains was confirmed for a subset of these samples, and most reassortants obtained a wild-type NS1 with a selective advantage. One isolate had an additional deletion of 185 nucleotides in its NS1 (14) and might therefore have had an increased virulence (15). Collectively, these data indicate safety concerns about the NS1del126-based Ingelvac Provenza LAIV and support the withdrawal of the vaccine from the market in 2020.

In summary, our results show that lvTX98 pathogenicity is less restricted than indicated by previous reports. Unlike *in vitro*, higher type I IFN induction by lvTX98 than by wtTX98 was not observed in swine lungs. lvTX98 attenuation *in vivo* is more likely caused by low NS1del126 protein stability and the loss of NS1 functions unrelated to IFN. We found considerable lvTX98 replication in the swine respiratory tract and substantial nasal excretion of lvTX98 by vaccinated pigs, which can facilitate reassortment with endemic IAV strains and reintroduction of the historic vaccine strains in the swine population. These data for Ingelvac Provenza H3N2 LAIV strain lvTX98 question the safety of NS1del126-based LAIVs.

## MATERIALS AND METHODS

### Ethics statement.

All animal experiments were approved by the Ethical and Animal Welfare Committee of the Faculty of Veterinary Medicine of Ghent University (project identification code, 2015-119; approval date, 14 December 2015).

### Cells and viruses.

PK-15 cells were grown in minimal essential medium (MEM with GlutaMAX; Gibco) supplemented with 10% fetal calf serum (FCS; Sigma) and antibiotics (100 IU/mL penicillin, 50 μg/mL streptomycin, 50 μg/mL gentamicin; Gibco). MDCK cells were cultured in MEM with 10% FCS, 1 mg/mL lactalbumin (BD Biosciences), and antibiotics. MDBK cells were grown in Dulbecco’s modified Eagle’s medium (DMEM with GlutaMAX; Gibco) supplemented with 10% FCS, 10 mM sodium pyruvate (Gibco), and antibiotics. B9 cells were maintained in Iscove’s modified Dulbecco’s medium (IMDM with GlutaMAX; Gibco) containing 5% FCS, 5 × 10^−5^ M β-mercaptoethanol (Sigma), 20 pg/mL recombinant human interleukin-6 (IL-6; R&D Systems), 100 IU/mL penicillin, and 50 μg/mL streptomycin. PK-15 subclone 15 cells were cultured in MEM with 7% FCS, 100 IU/mL penicillin, 50 μg/mL streptomycin, and 1% nonessential amino acids (Gibco).

Swine influenza A viruses A/swine/Texas/4199-2/1998 (wtTX98) and A/swine/Texas/4199-2/1998 NS1del126 (lvTX98) were generated via reverse genetics as previously described (15) and kindly provided by the National Animal Disease Center, Ames, IA, USA. lvTX98 has a 3′ deletion in the NS1 gene and produces a truncated NS1 protein consisting of only the first 126 amino acids. wtTX98 and lvTX98 swIAV stocks were grown in MDCK cells or in the allantoic cavity of 10-day-old embryonated chicken eggs. Vesicular stomatitis virus (VSV) for IFN-α/β bioassays was grown in MDBK cells.

### Infection of PK-15 cells.

Three- to 4-day-old PK-15 monolayers in 6-well plates were washed once with phosphate-buffered saline (PBS) with Ca^2+^ and Mg^2+^ and then infected with wtTX98 or lvTX98 at an MOI of 0.001 or an MOI of 2 in MEM supplemented with 0.6% bovine serum albumin (Sigma), 1 to 2 μg/mL trypsin (Sigma), and antibiotics (infection medium). After 1 h of incubation (37°C, 5% CO_2_), the unbound virus was washed away with PBS containing Ca^2+^ and Mg^2+^, 3.3 mL of infection medium was added per well, and 300 μL supernatant from each well was sampled immediately for virus and IFN-α/β titration. This time point corresponded to 0 hpi. The cells were then further incubated at 37°C and 5% CO_2_, and samples for virus and IFN-α/β titration were taken at different time points postinoculation by collecting 300 μL supernatant per well and then adding 300 μL fresh infection medium. Cells inoculated with an MOI of 0.001 were sampled at 0, 6, 10, 18, 24, 30, and 48 hpi. Cells inoculated with an MOI of 2 were sampled at 0, 2, 4, 6, 8, 10, 18, 24, 30, and 48 hpi. Five replicates of the experiment were performed.

### Infection of pigs.

Conventional 4-week-old pigs (*n* = 52) were purchased from a commercial farm that was free of influenza A virus. Pigs of different experimental groups were housed in separate high-efficiency particulate air (HEPA)-filtered biosafety level 2 animal units. At arrival, pigs were treated with a single dose of ceftiofur antibiotic (Naxcel; Zoetis) to reduce bacterial contamination. Pigs were confirmed to be seronegative in hemagglutination inhibition assay (34) for antibodies against swIAV representing endemic strains in Europe (A/swine/Belgium/1/1998 [H1N1], A/swine/Gent/172/2008 [H3N2], A/swine/Gent/7625/1999 [H1N2], and A/California/04/2009 [2009 pandemic H1N1]) at the start of the experiments.

To evaluate lvTX98 and wtTX98 excretion, 5-week-old pigs were intranasally inoculated with 6.3 log_10_ TCID_50_ of lvTX98 (*n* = 16) or wtTX98 (*n* = 6) in 3 mL Dulbecco’s PBS (DPBS; Gibco); 1.5 mL was applied in each nostril using a canula. At 0 to 7 dpi, pigs were monitored for clinical signs and nasal swabs (1 swab per nostril) were taken for virus titration and sequencing. Pigs were then further used in another experiment. Because of the experimental design of the latter, different numbers of pigs were inoculated with lvTX98 versus wtTX98. Afterward, all pigs were humanely euthanized with a lethal dose of sodium pentobarbital (Kela NV).

To examine the pathogenesis of lvTX98 and wtTX98, 2 groups of 15 6-week-old pigs were used. In each group, 3 pigs were left noninoculated and were humanely euthanized with a lethal dose of sodium pentobarbital at 0 hpi. The 12 remaining pigs were inoculated intratracheally with 7.5 log_10_ TCID_50_ of lvTX98 or wtTX98 in 3 mL DPBS; inocula contained <1.32 endotoxin units per mL in the *Limulus* assay (Lonza). At 18, 24, 48, and 72 hpi, 3 inoculated pigs per group were humanely euthanized with a lethal dose of sodium pentobarbital. From 4 days before inoculation until the end of the experiment, each pig was scored for clinical signs according to the scoring system shown in Table 1, and a composite score was calculated for each pig at each time point (−96, −72, −48, −24, 0, 18, 24, 48, and 72 hpi). At each time point of euthanasia (0, 18, 24, 48, and 72 hpi), nasal swabs were collected (1 swab per nostril) for virus titration and sequencing. At necropsy, lungs were scored for macroscopic lesions and different parts of the respiratory tract were sampled for virus titration, histopathology, sequencing, and cytokine analysis. Virus titration was performed on samples from trachea, nasopharynx, nasal mucosa olfactory part, nasal mucosa respiratory part, left apical and cardiac lung lobe, and left diaphragmatic lung lobe. Histopathological analysis was performed on a sample from trachea and left diaphragmatic lung lobe. The right lung was used to collect BAL fluid for BAL cell and cytokine analysis, virus titration, and sequencing.

### BAL and BAL cell analysis.

The right lung was flushed with 80 mL of cold DPBS using a blunt 18-gauge needle inserted through the trachea. BAL fluids (42 to 60 mL) were centrifuged for 10 min at 400 × *g* and 4°C to separate the cells from cell-free BAL fluid. BAL cells were resuspended in DPBS, and their number and viability were determined using Türk’s solution and trypan blue. Cytocentrifuge preparations (2 per pig) were made with 300 μL of a BAL cell suspension of 5 × 10^5^ cells/mL and stained with DiffQuik (Medion Diagnostics) to determine the number of neutrophils and macrophages. Forty milliliters of cell-free BAL fluid from each pig was ultracentrifuged for 90 min at 100,000 × *g* to remove virus, after which it was concentrated 20 times using Centricon Plus-70 centrifugal filter units with a cutoff of 10 kDa (Millipore). This concentrated BAL fluid was used for cytokine analysis. The leftover cell-free BAL fluid was used for virus titration and sequencing.

### Pathological examination of lungs.

The percentage of gross pneumonia was determined by visual inspection of the lung surface, and tissue samples for histopathological examination of microscopic lesions were processed and scored as previously described (12).

### Virus titration.

The 2 rayon nasal swabs collected from each pig were placed together in 1 mL PBS with Ca^2+^ and Mg^2+^ supplemented with 10% FCS, 1,000 IU/mL penicillin, and 500 μg/mL streptomycin and shaken for 1 h at 4°C. The medium was then collected and clarified via centrifugation (2 min, 13,000 rpm, 4°C). Tissue samples were homogenized in PBS with Ca^2+^ and Mg^2+^ to obtain 20% suspensions and clarified via centrifugation (20 min, 2,000 rpm, 4°C). Virus stocks for inoculation, supernatants of PK-15 cells, and cell-free BAL fluid were used as such. Virus titration was performed via cytopathic effect (CPE) assay on MDCK cells (35), and virus titers were calculated using the formula of Reed and Muench (36).

### Cytokine analysis.

Bioassays for the detection of IFN-α/β, IL-6, and TNF-α were performed as previously described (19). Levels of bioactive IFN-α/β were determined using a CPE reduction assay with VSV on MDBK cells. A porcine recombinant IFN-α solution (gift from INRA, France) with a titer of 12.8 log_2_ U/mL was tested in each assay as a laboratory standard. Supernatants of infected PK-15 cells were tested once; the detection limit was 6.3 log_2_ U/mL. For 20-times-concentrated BAL fluids, each result represents the mean from 2 (IFN-α versus IFN-β) or 3 (IFN-α/β) independent tests with a detection limit of 4.3 log_2_ U/mL. Bioactive IL-6 was quantitated via its ability to stimulate B9 cell proliferation. A recombinant human IL-6 solution (R&D Systems) with a titer of 13.4 log_2_ U/mL was tested in each assay as a laboratory standard. All samples were tested in duplicate in 2 independent assays with a detection limit of 5.3 log_2_ U/mL, and results are represented as the mean titers from the 2 independent assays. Bioactive TNF-α titers were determined using a cytotoxicity assay on PK-15 subclone 15 cells. A porcine recombinant TNF-α solution (R&D Systems) with a titer of 14.6 log_2_ U/mL was tested in each assay as a laboratory standard. Each result represents the mean from 3 independent tests with a detection limit of 5.3 log_2_ U/mL.

Enzyme-linked immunosorbent assay (ELISA) kits used for detection of IFN-α and IFN-β in 20-times-concentrated BAL fluids were the Verikine-HS pig interferon alpha ELISA kit from PBL Assay Science (catalog no. 47100-1, lot no. 7031, detection range of 2.34 to 150 pg/mL) and the porcine IFN-β ELISA kit from Emelca Bioscience (catalog no. MBS760799-2, lot no. P0074E040, detection range of 15.625 to 1,000 pg/mL). ELISAs were performed on 1/100-prediluted samples according to manufacturer’s instructions.

### Sequencing.

Whole-genome sequencing of lvTX98 and wtTX98 viruses in virus stocks, nasal swab samples of intranasally inoculated pigs, and cell-free BAL fluids of intratracheally infected pigs was performed by using MinION (Oxford Nanopore Technologies) as previously described (37). wtTX98 sequences were downloaded from GenBank (accession numbers CY095672 to CY095679) and used as reference sequences.

### Statistical analysis.

Mean log_10_-transformed virus titers and log_2_-transformed cytokine titers for each virus at each time point were calculated by assigning negative samples a value corresponding to half the value of the detection limit. For viral growth and IFN-α/β induction in PK-15 cells, the area under the curve was calculated for each replicate of the experiment. Results for lvTX98 and wtTX98 were compared using two-sided Mann-Whitney U tests. *P* values of <0.05 were considered significant, except when only 3 observations per group were available (data for euthanized i.t.-inoculated pigs), and then *P* values of ≤0.1 were considered significant. All analyses were performed using R version 3.2.2 (38).
